# Administration of Corticosteroids for Prompt Suppression of Cytokine Storm in Severe Cases of Japanese Spotted Fever

**DOI:** 10.7759/cureus.67857

**Published:** 2024-08-26

**Authors:** Narumichi Iwamura, Kanako Tsutsumi, Takafumi Hamashoji, Yui Arita, Takashi Deguchi

**Affiliations:** 1 Department of Nephrology, Yamaguchi Red Cross Hospital, Yamaguchi, JPN

**Keywords:** interleukin-6 amplifier, tick-borne disease, corticosteroid, disseminated intravascular coagulation, cytokine storm syndrome, japanese spotted fever

## Abstract

Japanese spotted fever (JSF) is a tick-borne disease caused by *Rickettsia japonica*and primarily affects the warmer coastal areas of Japan. Early treatment with tetracycline antibiotics is crucial to prevent severe complications, such as pneumonia, meningitis, disseminated intravascular coagulation (DIC), and systemic inflammatory response syndrome. An 83-year-old man with hypertension, chronic kidney disease, and hyperuricemia presented with DIC and subsequently developed septic shock. Polymerase chain reaction confirmed JSF caused by *R. japonica*. Initial treatment with ceftriaxone was ineffective, leading to a switch to intravenous minocycline and levofloxacin. Considering the high levels of C-reactive protein, procalcitonin, ferritin, and soluble interleukin-2 receptor, intravenous hydrocortisone (200 mg/day) was administered to control the cytokine storm. On day 4, the patient’s condition improved significantly, with increased blood pressure, reduced DIC markers, and decreased levels of inflammatory cytokines, including interleukin-6 and tumor necrosis factor-α. The patient’s recovery continued, and he was transferred to a chronic care hospital. Severe JSF cases are primarily driven by a cytokine storm caused by an excessive immune response. Early administration of corticosteroids along with antibiotics effectively suppressed the cytokine storm in this case. Reports have shown mixed results, indicating the need for further research to determine the optimal type, dosage, and duration of corticosteroid treatment.

## Introduction

Japanese spotted fever (JSF) is a disease caused by *Rickettsia japonica*, first documented in Japan in 1984 [1]. It primarily affects the warmer coastal areas in the southwestern and central parts of Japan [2]. JSF shares symptoms with other tick-borne diseases, including high fever, a rash, and an eschar at the tick bite site [3]. Early antibiotic treatment with tetracycline is crucial; without it, patients risk developing severe complications, such as pneumonia, meningitis, disseminated intravascular coagulation (DIC), and systemic inflammatory response syndrome (SIRS), potentially leading to multiple organ failure [4]. We encountered a severe case of JSF in an 83-year-old man who presented with DIC and subsequently developed septic shock. To address the presumed high cytokine levels, we administered corticosteroids along with antibiotics to control the cytokine storm, resulting in the patient’s survival. Because of concerns about immunosuppression, few physicians have used corticosteroids in severe cases of JSF. In addition to routine blood tests, we measured interleukin (IL)-6 and tumor necrosis factor-α (TNF-α) before and after corticosteroid administration and reported the following findings.

## Case presentation

An 83-year-old man with hypertension, chronic kidney disease due to hypertensive nephrosclerosis, and hyperuricemia presented to our hospital. The patient’s serum creatinine (sCr) levels and estimated glomerular filtration rate (eGFR) on May 18, 2024, were 1.81 mg/dL and 28.4 mL/min/1.73 m^2^, respectively. The patient was taking a combination of candesartan cilexetil (8 mg/day) and amlodipine (5 mg/day) for hypertension and allopurinol (100 mg/day) for hyperuricemia. He had no history of smoking and occasionally consumed alcohol. On June 1, 2024, the patient went bamboo shoot digging in a bamboo grove but did not notice any insect bites, including tick bites. On June 6, 2024, he began to feel general fatigue, and from June 7, 2024, he was so exhausted that he could not get out of bed or eat. On June 9, 2024, he experienced diarrhea with bloody stools. A blood test performed by his primary care physician on June 11, 2024, revealed a significant increase in C-reactive protein (CRP) levels of 19.1 mg/dL and impaired kidney function (sCr of 5.53 mg/dL and eGFR of 8.38 mL/min/1.73 m^2^), leading to an emergency transport to our hospital on the same day.

Upon admission, the patient was fully conscious (Glasgow coma scale score of 15), with a body temperature of 38.3°C, blood pressure of 98/53 mmHg, pulse rate of 90/min, and SpO_2_ of 97% on room air. Petechial rashes were observed on his trunk and extremities, with no evidence of coalescence. The lymph nodes were palpable in the left neck and both inguinal regions. Blood tests revealed thrombocytopenia (platelet count of 55,000/μL), a mild increase in lymphocyte count (9,200/μL), significantly elevated D-dimer levels (32.8 μg/mL), highly elevated fibrinogen degeneration products (FDPs; 77.6 μg/mL), elevated liver enzymes (aspartate aminotransferase (AST) of 128 U/L, aminotransferase (ALT) of 77 U/L, alkaline phosphatase (ALP) of 544 U/L, γ-glutamyl transpeptidase (γ-GTP) of 119 U/L, increased sCr (5.53 mg/dL), decreased eGFR (8.38 mL/min/1/73 m2), high ferritin (3,611 ng/mL), high CRP (19.1 mg/dL), elevated procalcitonin (27.62 ng/mL), and elevated soluble IL-2 receptor (sIL-2R). These findings suggest severe inflammation accompanied by DIC, acute liver injury, and acute kidney injury. Urinalysis revealed hematuria (>100/HPF), urinary protein-to-urinary creatinine ratio (UPCR) of 1.11 g/gCr, and elevated levels of β2-microglobulin (3195 μg/L), α1-microglobulin (83.9 MG/L), and β-N-acetyl-glucosaminidase (103.5 IU/L), indicating severe tubular injury (Table 1).

**Table 1:** Laboratory test results at admission, on day 2, on day 4, and at discharge PT-INR: Prothrombin time-international normalized ratio; PT: Prothrombin time; APTT: Activated partial thromboplastin time; eGFR: Estimated glomerular filtration rate; IL-2: Interleukin-2; PR3-ANCA: Proteinase 3-antineutrophil cytoplasmic antibodies; MPO-ANCA: Myeloperoxidase-antineutrophil cytoplasmic antibodies; GBM: Glomerular basement membrane.

	Reference range	Day 1	Day 2	Day 4	Day 27
Blood cell counting					
Hemoglobin	13.7–16.8 g/dL	11.3	11.5	9.3	10
Hematocrit	40.7%−50.1％	32	31.8	26.4	30.3
Platelet	158–348 k/μL	55	51	64	307
White blood cells	3.30–8.60 μL	9.2	9.4	10.9	4.9
Neutrophil	38.0%–58.9％	85.9	87.4	82.4	44.4
Lymphocyte	20.0%–40.0％	11.8	10.5	14.9	39.6
Eosinophil	1.0%–4.0％	0	0	0	3
Coagulation					
PT-INR		0.82	0.82	1.01	0.83
PT	10.7–12.9 s	10.9	10.9	13.2	10.9
APTT	24.0–38.0 s	41.6	38.5	34.2	27.6
D-dimer	0.0–1.0 μg/mL	32.8	47.2	14.1	14.8
Fibrin degradation products	0.0–5.0 μg/mL	77.6	107.8	N/A	N/A
Fibrinogen	150–350 mg/dL	203	173	N/A	N/A
Chemistry					
Total protein	6.6–8.1 g/dL	5.8	5.6	5.5	6.4
Albumin	4.1–5.1 g/dL	2.3	2.1	2.1	2.8
Aspartate aminotransferase	13–30 U/L	128	117	77	21
Alanine aminotransferase	10–42 U/L	77	68	60	12
Lactate dehydrogenase	124–222 U/L	377	377	294	190
Alkaline phosphatase	38–113 U/L	544	558	391	148
γ-Glutamyl transpeptidase	13.0–64 U/L	119	138	120	38
Amylase	44–132 U/L	132	138	289	244
Creatine kinase	43–270 U/L	419	295	53	17
Creatinine	0.65–1.07 mg/dL	5.53	4.61	3.29	1.12
eGFR	>90 ml/min/1.73 m^2^	8.4	10.3	14.8	48.2
Uric acid	3.70–7.80 mg/dL	10.9	10.6	8.4	6.6
Urea nitrogen	8.0–20 mg/dL	137.4	134.8	125.5	15.2
Sodium	138–145 mEq/L	130	134	144	142
Potassium	3.6–4.8 mEq/L	4.8	4.4	4.3	3.3
Chlorine	101–108 mEq/L	94	101	113	106
Total bilirubin	0.400–1.50	0.6	0.6	0.3	0.4
Hemoglobin A1c	4.9–6.0 mg/dL	5.9	N/A	N/A	N/A
Blood sugar	73%–109％	145	105	143	85
Ferritin	21.8–274.7 ng/mL	3611	N/A	N/A	113
Immunology					
C-reactive protein	0.00–0.140 mg/dL	19.1	13.9	5.72	1
Procalcitonin	<0.05 ng/mL	27.6	N/A	N/A	0.09
Soluble IL-2 receptor	157–474 U/mL	10500	N/A	N/A	1160
Complement component 3	73–138 mg/dL	106	N/A	N/A	N/A
Complement component 4	11–31 mg/dL	41	N/A	N/A	N/A
50% hemolytic nit of complement	31.6–57.6 U/mL	57.3	N/A	N/A	N/A
Antinuclear antibody	<40 titers	<40	N/A	N/A	N/A
Immunoglobulin G	861−1747 mg/dL	1142	N/A	N/A	N/A
Immunoglobulin A	93−393 mg/dL	518	N/A	N/A	N/A
Immunoglobulin M	33−183 mg/dL	84	N/A	N/A	N/A
PR3-ANCA	<3.5 U/mL	<1.0	N/A	N/A	N/A
MPO-ANCA	<3.5 U/mL	<1.0	N/A	N/A	N/A
Anti-GBM antibody	<3.0 U/mL	<2.0	N/A	N/A	N/A
κ/λ ratio	0.26–1.65	0.31	N/A	N/A	1.03
Urinary					
β2-microglobulin	14–329 ng/mL	3195	N/A	N/A	N/A
α1-microglobulin	1.0–15.5 mg/L	83.9	N/A	N/A	N/A
N-acetyl-β-D-glucosaminidase	0.7–11.2 IU/L	103.5	N/A	N/A	N/A
Urinary protein/Urinary creatinine	<0.05 g/gCr	1.11	1.38	N/A	0.49
Urinary blood	0–4/high-power field	>100	50−90	N/A	10–19
Serum cytokine					
Interleukin-6	<7.0 pg/mL	N/A	69.8	4.1	N/A
Tumor necrosis factor-α	0.75−1.66 pg/mL	N/A	26.5	14.7	N/A

**Figure 1:**
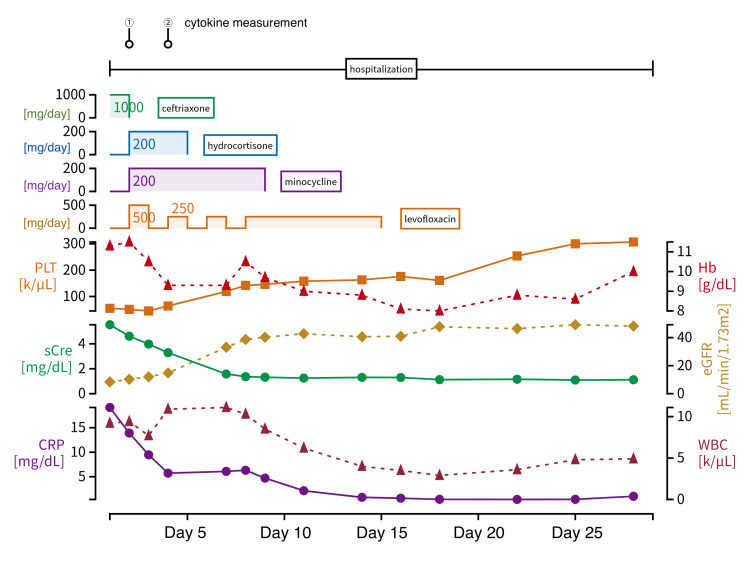
Clinical course PLT: Platelet; sCr: Serum creatinine; CRP: C-reactive protein; Hb: Hemoglobin; eGFR: Estimated glomerular filtration rate; WBC: White blood cells.

Chest and abdominal computed tomography revealed no significant abnormalities. We have presented the patient’s course after hospitalization in Figure 1.

On day 1, the patient was intravenously administered ceftriaxone (1 g/day) for bacterial infection of unknown origin. Over time, the patient’s blood pressure decreased. Despite appropriate fluid resuscitation, norepinephrine was necessary to maintain a mean arterial blood pressure of 65 mmHg, leading to the diagnosis of septic shock. On day 2, the patient’s platelet count decreased from 55,000/μL to 51,000/μL, D-dimer levels increased from 32.8 μg/mL to 47.2 μg/mL, and FDP levels increased from 77.6 μg/mL to 107.8 μg/mL, indicating the progression of DIC. Because of poor response to antibiotics, a thorough re-evaluation was performed, revealing a 3-mm eschar on the left buttock. Considering the triad of fever, rash, and eschar, tick-borne diseases, particularly scrub typhus or JSF, were suspected. Consequently, on day 2, the antibiotic regimen was changed to intravenous minocycline (200 mg/day) and levofloxacin (initially 500 mg/day, followed by 250 mg/day every other day). Considering the patient’s elevated CRP, procalcitonin, ferritin, and sIL-2R levels, it was considered that the patient was in a state of high inflammatory response or SIRS. Therefore, to suppress the cytokine storm, intravenous administration of hydrocortisone (200 mg/day) for three days was initiated on day 2. On day 4, the patient’s blood pressure increased over time, allowing the discontinuation of norepinephrine, suggesting he overcame septic shock. Blood tests revealed an increase in platelet count from 51,000/μL to 64,000/μL, a decrease in D-dimer levels from 47.2 μg/mL to 14.1 μg/mL, and a reduction in CRP levels from 13.9 mg/dL to 5.72 mg/dL, suggesting improvement in DIC and inflammatory response.

Polymerase chain reaction conducted on the eschar from the left buttock and blood test on day 2 were positive for *R. japonica* 17 kDa, indicating JSF caused by *R. japonica*. During the course, amylase levels gradually increased, peaking at 1,406 U/L on day 8. Suspecting acute pancreatitis caused by minocycline, the antibiotic treatment was switched to levofloxacin monotherapy on day 9. Subsequently, laboratory findings, including the inflammatory response and systemic condition, improved over time, and antibiotic treatment was completed on day 15. After continued dietary therapy for acute pancreatitis, the patient was transferred to a chronic care hospital on day 27. On day 27, the platelet count increased to 307,000/μL, and liver enzymes normalized (AST: 21 U/L; ALT: 12 U/L; ALP: 38 U/L; γGT: 38 U/L). SCr levels improved to 1.12 mg/dL and eGFR to 48.2 mL/min/1.73 m^2^, and urinary findings revealed UPCR of 0.49 g/gCr and urine occult blood of 10-10/HPF. CRP levels decreased to 1 mg/dL, procalcitonin (PCT) to 0.09 ng/mL, and sIL-2R to 1,160 U/mL (Table 1). The plan was to continue follow-up and rehabilitation for chronic kidney disease, with the aim of returning the patient to the chronic care hospital for home-based care.

## Discussion

The incubation period for JSF is two to eight days. Similar to scrub typhus, JSF is clinically characterized by fever, rash, and eschar, which are collectively termed the triad of JSF. The fever can reach approximately 40°C, presenting as remittent fever. The rash appears as irregularly shaped erythema ranging in size from 4 to 9 mm, sometimes becoming hemorrhagic, and is predominantly found on the extremities, including the face, palms, and soles. The eschar is relatively small, with a detection rate of approximately 60%-70%, and tends to be smaller than that observed in scrub typhus [5]. JSF symptoms include headache, high fever, chills, sudden onset, general fatigue, joint pain, and muscle pain. Pharyngitis is also common, and lymphadenopathy is less prominent in JSF than in scrub typhus. Gastrointestinal disturbances, particularly acute gastric mucosal lesions, gastric ulcers, and duodenal ulcers, are frequently observed. According to the infectious disease surveillance report (n = 1,765:2007-2016), fever occurred in 99% of cases, rash in 94%, liver function abnormalities in 73%, eschar in 66%, headache in 31%, and DIC in 20%. Severe cases may exhibit signs of DIC, including thrombocytopenia, with abnormal test values being more pronounced than those in scrub typhus [5]. In this case, the patient developed symptoms after an incubation period of approximately five days, presenting with a fever above 38°C, irregularly bordered petechial rash on the extremities and trunk, and a small 3-mm eschar on the left buttock. Upon arrival, the patient had a high fever, chills, and general fatigue. Later, hematochezia developed, which was found to be caused by acute gastric mucosal lesions on upper gastrointestinal endoscopy. In general, JSF presents with strongly positive CRP and elevated liver transaminase levels, although lymphocyte count changes are inconsistent. Although the patient’s CRP level was high at 19.1 mg/dL upon admission, liver enzymes were not elevated and the lymphocyte count was mildly elevated at 9,200/μL. The patient was diagnosed with DIC based on decreased platelet count and elevated FDP and D-dimer levels.

In this severe case of JSF, which was complicated by DIC and progressed to septic shock, corticosteroids were administered in the initial treatment to suppress the cytokine storm early. As detailed in the "Case Presentation" section of this article, hydrocortisone was added to the regimen of minocycline and levofloxacin on day 2. On day 4, the inflammatory response had decreased, the patient had overcome septic shock, and the DIC had shown signs of improvement. Therefore, combination therapy of minocycline, levofloxacin, and hydrocortisone was clinically effective in this severe case of JSF. In our research, only four cases have been reported, including this one, in which corticosteroids were used along with minocycline to treat severe JSF complicated by DIC (Table 2) [6-8].

**Table 2:** Summary of Japanese spotted fever cases with corticosteroids DIC: Disseminated intravascular coagulation; ISTH: International Society on Thrombosis and Haemostasis.

Items	Reference range	Iwasaki et al. [6]	Wada et al. [7]	Takiguchi et al. [8]	Present case
Year of onset	Years	1998	2004	2013	2024
Age	Years	78	77	70	83
Female/male		F	F	F	M
Strain of rickettsia		R. japonica	R. japonica	R. japonica	R. japonica
Days from onset to the first visit		3	1	5	3
Peak of C-reactive protein	0.00–0.30 mg/dL	17.5	24.6	16.8	19.13
Nadir of platelet counts	1.5–4.0 ×10^4^/μL	7.4	3.8	3.6	4.5
Fibrinogen degradation products	0.0–5.0 μg/mL	40	29.8	N/A	77.6
D-dimer	0.0–1.0 μg/dL	N/A	29.6	65.3	32.8
Japanese criteria for acute-phase DIC [9]	3 or less	Yes (6 or above)	Yes (7 or above)	Yes (7 or above)	Yes (6)
ISTH criteria for DIC diagnosis [10]	4 or less	Yes or no (4 or above)	Yes (5 or above)	Yes (5 or above)	Yes (4)
Antibiotic treatment		200 mg/day of minocycline and 300 mg/day of ciprofloxacin	200 mg/day of minocycline and 300 mg/day of ciprofloxacin	200 mg/day of minocycline and 500 mg/day of levofloxacin	200 mg/day of minocycline and 500 mg/day of levofloxacin
Administration method of corticosteroids		1000 mg/day of methylprednisolone on days 1–3	500 mg/day of methylprednisolone on days 2–3	1000 mg/day of methylprednisolone on days 4–6, following 50 mg/day of prednisolone	200 mg/day of hydrocortisone on days 2–4
Outcome		Recovery	Death on day 3	Recovery	Recovery

The sex ratio of these cases was three females and one male, with a median age at onset of 77.5 years. All cases were diagnosed with JSF caused by *R. japonica* via genetic testing and met the Japanese criteria for acute-phase DIC [9]; all but one case met the International Society on Thrombosis and Haemostasis criteria for DIC diagnosis [10]. The median peak CRP level was 18.3 mg/dL, indicating a significant inflammatory response. All patients were treated with antibiotics, particularly minocycline (200 mg/day) combined with either ciprofloxacin (300 mg/day) or levofloxacin (500 mg/day). Iwasaki et al. [6] reported patient recovery after three days of intravenous methylprednisolone (1,000 mg/day). Meanwhile, Wada et al. administered intravenous methylprednisolone (500 mg/day) for two days but could not save the patient [7]. Takiguchi et al. assumed collagen vascular disease-associated interstitial lung disease with systemic sclerosis and administered intravenous methylprednisolone (1,000 mg/day) for three days, followed by oral PSL (50 mg/day), resulting in patient recovery [8]. In this case, intravenous hydrocortisone (200 mg/day) administered for three days led to immediate recovery from septic shock, improvement in DIC, and reduction in the inflammatory response, ultimately saving the patient.

To date, the use of corticosteroids along with minocycline for JSF has been limited, with three of four cases resulting in survival. The administration of corticosteroids was expected to have positive effects, such as improving relative adrenal insufficiency, exerting anti-inflammatory and vasoconstrictive actions, and enhancing responsiveness to vasopressors. On the other hand, there were concerns about increased risks of complications such as infections due to immunosuppression, gastrointestinal bleeding, and hyperglycemia. The 2024 Japanese guidelines for septic shock, which recommend low-dose hydrocortisone (200-300 mg/day) for patients with septic shock who do not respond to initial fluid resuscitation and vasopressor therapy, were considered [11]. Clinical experience indicates that corticosteroids are an effective treatment for cytokine storm syndrome, with a rapid steroid taper typically achievable within several days without recurrence of the condition. Although the dosing and choice of corticosteroid should be individualized, a commonly used initial dose is methylprednisolone at 2 mg/kg/day, which can generally be tapered over several days [12]. Taking into account the risk-benefit balance and previous reports of successful treatment of severe JSF with corticosteroids, we decided to administer hydrocortisone (200 mg/day) for three days primarily to mitigate the cytokine storm.

According to Tai et al. [13], cytokine and chemokine levels during the acute phase of JSF are significantly higher than those observed during the acute phase of Tsutsugamushi disease [14]. The uncontrolled excessive systemic release of numerous cytokines and chemokines in life-threatening infections can lead to systemic dysfunction, potentially resulting in fatal outcomes, such as septic shock and SIRS [14,15]. In general, despite antibiotic treatment, the clinical course of JSF is considered worse than that of Tsutsugamushi disease. Patients with severe JSF who experience complications and do not respond to monotherapy with minocycline or doxycycline may be successfully treated with a combination of tetracyclines and quinolones (e.g., ciprofloxacin or levofloxacin) [16].

To investigate the impact of combined antibiotic and corticosteroid therapy on cytokine profiles, we measured IL-6 and TNF-α levels on day 2 before the administration of antibiotics and hydrocortisone and again 48 h later on day 4 after the administration of the antibiotics and corticosteroids (Table 1). On day 2, the IL-6 level was 69.8 pg/mL and TNF-α was 26.5 pg/mL. After 48 h of intravenous administration of minocycline (200 mg/day), levofloxacin (500 mg/day), and hydrocortisone (200 mg/day), the IL-6 level decreased to 4.1 pg/mL (a reduction of 65.7 pg/mL), and TNF-α decreased to 14.7 pg/mL (a reduction of 11.8 pg/mL). Tai et al. examined 21 patients with JSF who were not treated with corticosteroids and examined cytokine profile changes in both the acute (at the first consultation) and recovery (two weeks later) phases. Their findings revealed that the median IL-6 level was 65.8 pg/mL in the acute phase and 2.2 pg/mL in the recovery phase, indicating a decrease of 63.6 pg/mL over two weeks. Meanwhile, the median TNF-α level was 35.0 pg/mL in the acute phase and 6.8 pg/mL in the recovery phase, showing a decrease of 28.2 pg/mL over two weeks [13]. Although direct comparisons are not ideal, it is possible that the decrease in IL-6 and TNF-α levels achieved within two weeks without corticosteroids was reached within 48 h with antibiotics and the addition of intravenous hydrocortisone (200 mg/day).

The mechanism by which high cytokine levels persist in patients with JSF despite the administration of sufficient amounts of effective antibiotics, such as minocycline and levofloxacin, is not well understood. We speculate that one potential mechanism involves an IL-6 amplifier (IL-6 Amp). Activated CD4+ cells and other cells release cytokines that simultaneously activate the nuclear factor kappa-light-chain enhancer of activated B cells (NF-κB) and signal transducer and activator of the transcription 3 (STAT3) pathways in nonimmune cells, such as fibroblasts and endothelial cells. This activation boosts NF-κB signaling, resulting in the local production of large amounts of chemokines, growth factors, IL-6, and other inflammatory cytokines. This mechanism, called the “IL-6 Amp,” leads to the IL-6 produced in this pathway, further activating STAT3 resulting in further production of IL-6 and thereby sustaining the IL-6 Amp. The inflammatory cytokines, chemokines, and growth factors produced through this pathway attract various inflammatory cells and increase cytokine secretion. These cytokines stimulate local cell proliferation, further amplifying the IL-6 Amp, which can result in chronic inflammation and the onset of a cytokine storm. IL-6 Amp can occur under conditions where IL-6 activates STAT3 and TNF-α, IL-1, or IL-17, leading to the adequate activation of NF-κB [17,18]. In this case, IL-6 and TNF-α levels on day 2 were high enough to trigger the IL-6 Amp. Therefore, we speculate that the high inflammatory response and DIC observed in this patient were driven by a cytokine storm caused by the IL-6 Amp. As severe JSF cases are believed to be primarily caused by a cytokine storm caused by an excessive immune response in the host, early administration of corticosteroids along with antibiotics may be effective in suppressing the cytokine storm.

## Conclusions

In this severe case of JSF complicated by DIC and septic shock, early intravenous administration of hydrocortisone (200 mg/day) along with minocycline and levofloxacin effectively suppressed the cytokine storm. Our findings suggest that early intervention with corticosteroids, along with appropriate antibiotic therapy, improves outcomes in severe JSF cases by controlling excessive inflammatory responses. However, further research is required to determine the optimal type, dosage, and duration of corticosteroid treatment along with antibiotics for managing severe JSF.
